# Surgical and Medical Co‐Management in an 82‐Year‐Old Patient With Hemophilia a Undergoing Pancreaticoduodenectomy

**DOI:** 10.1002/hcs2.70016

**Published:** 2025-05-25

**Authors:** Wenning Lu, Chaoyang Liu, Jing He, Rui Cheng

**Affiliations:** ^1^ Department of Comprehensive Surgery, The Second Medical Center & National Clinical Research Center for Geriatric Diseases Chinese PLA General Hospital Beijing China

**Keywords:** co‐management, hemophilia A, pancreaticoduodenectomy

## Abstract

We report the successful application of a surgical and medical co‐management (SMC) strategy in an 82‐year‐old man with hemophilia A (HA) undergoing pancreaticoduodenectomy for pancreatic head carcinoma. No major complications or perioperative bleeding occurred. Optimal management of HA patients undergoing major surgery requires multidisciplinary coordination to avoid postoperative complications. The SMC team integrates internists (who assess chronic disease status, adjust medications, and determine best hemostatic therapies) and surgeons (who evaluate the surgical feasibility of procedures and rely on advanced surgical skills) to improve perioperative planning to minimize complications and promote recovery. This case illustrates the utility of a shift from passive and conservative treatment to active and preventive treatment and highlights the value of SMC in many complex clinical situations.

## Graphical abstract

The surgical and medical co‐management (SMC) team integrates internists and surgeons to improve perioperative planning to minimize complications and promote recovery. SMCs team implemented various perioperative optimization strategies for the patient to prevent complications and enhance satisfaction. This case illustrates the utility of a shift from passive and conservative treatment to active and preventive treatment and highlights the value of SMC in many complex clinical situations.

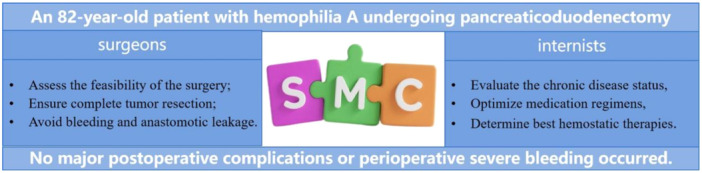

**Abbreviations**
HA: hemophilia A
PD: pancreaticoduodenectomy
RPD: robotic assisted pancreaticoduodenectomy
SMC: surgical and medical co‐management

## 1 Background

Pancreaticoduodenectomy (PD) is one of the most challenging abdominal surgeries, with high risks of mortality from intraoperative bleeding and postoperative complications [1]. Hemophilia A (HA), a rare congenital bleeding disorder, poses significant surgical challenges as some patients may develop severe or catastrophic bleeding after highly invasive surgery [2]. Moreover, cases of octogenarians with HA undergoing PD are rare, and limited literature exists to evaluate management in such scenarios. Optimal care of people with hemophilia, especially for those undergoing surgeries that carry a high risk of bleeding, requires comprehensive care provided by a multidisciplinary team of specialists. Surgical and medical co‐management (SMC) is a new strategy for optimizing the treatment outcomes of chronic disease patients undergoing complex surgeries [3, 4].

We present what we believe to be the first reported case of SMC strategy in the treatment of an 82‐year‐old man with HA who successfully underwent PD surgery.

## 2 Case Presentation

**Figure 1:**
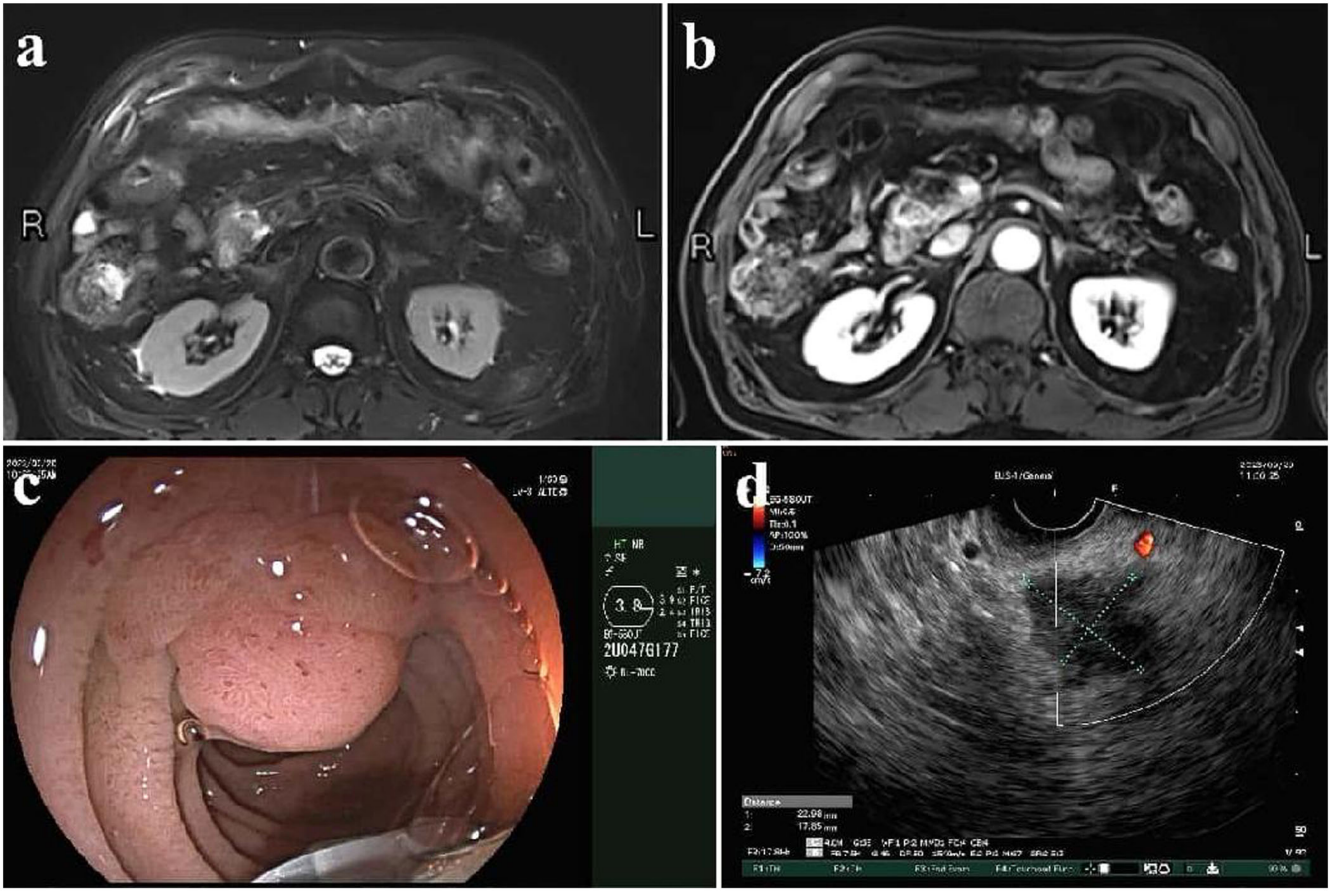
MRI and endoscopic ultrasound showed a mass at pancreatic head. (a) MRI scan; (b) enhanced MRI scan; (c) endoscopic show external compression of the tumor on the duodenal wall; (d) endoscopic ultrasound suggests invasive tumor.

An 82‐year‐old male patient presented with a diagnosis of pancreatic head cancer following a 3‐month history of paroxysmal upper abdominal pain without jaundice or fever. Imaging with abdominal MRI (Figure 1a,b) and endoscopic ultrasound(Figure 1c,d) showed a 2.3 cm × 1.8 cm mass in the pancreatic head. Bilirubin at admission was normal. His medical history included mild HA (diagnosed after excessive bleeding post‐dental extraction 20 years prior), hypertension, arrhythmia, and a previous laparoscopic cholecystectomy. Coagulation studies showed factor VIII activity at 21% and a significantly prolonged activation of the partial thromboplastin time.

Upon admission, the SMC team provided preoperative counseling and psychological support and discussed the risks and benefits of his treatment options. Factor VIII (Novo Nordisk, NJ, USA) was administered intravenously 24 h before surgery, increasing his factor VIII activity from 30% to 60% on the morning of surgery. Particular attention was taken regarding hemostasis through transfusion of six units of cryoprecipitate during the 6‐h procedure.

**Figure 2:**
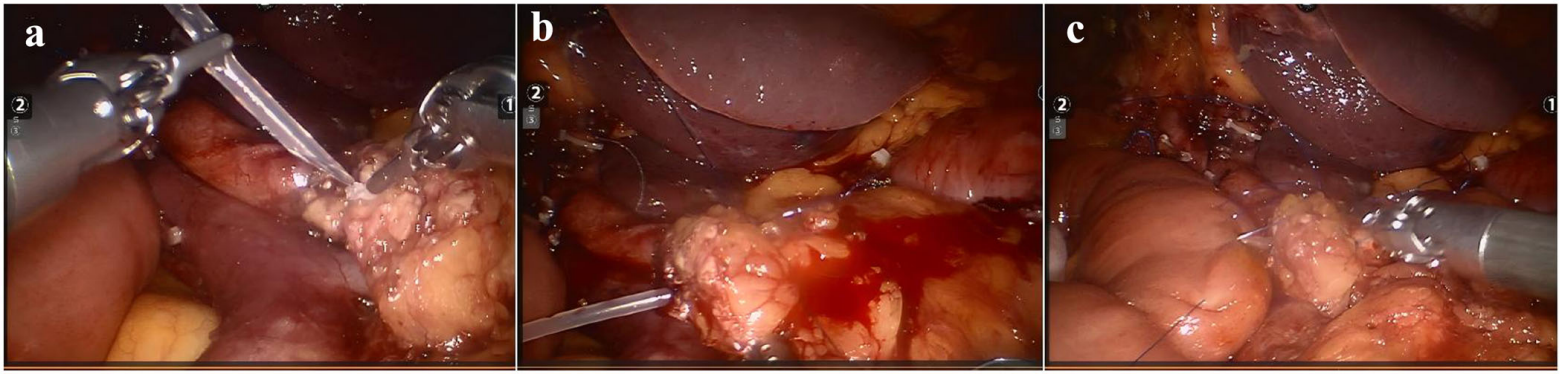
The key steps followed in a RPD performed for a technically to reduce the incidence of pancreatic fistula. (a) An internal stent is inserted into the main pancreatic duct fixed with 5.0 Vicry1; (b) two horizontal figure‐eight sutures were places through the full thickness of the pancreas; (c) single layer of the continuous suturing between the pancreas and the jejunum was performed using Prolene 4‐0.

The patient underwent minimally invasive robotic‐assisted pancreaticoduodenectomy (RPD), which has been previously described in detail [5]. Figure 2a–c demonstrates the key steps followed in the RPD to reduce the chance of a pancreatic fistula and highlights the robotic control of bleeding. Total anesthesia time was 350 min with robotic operative time of 240 min; estimated blood loss was 100 mL.

**Figure 3:**
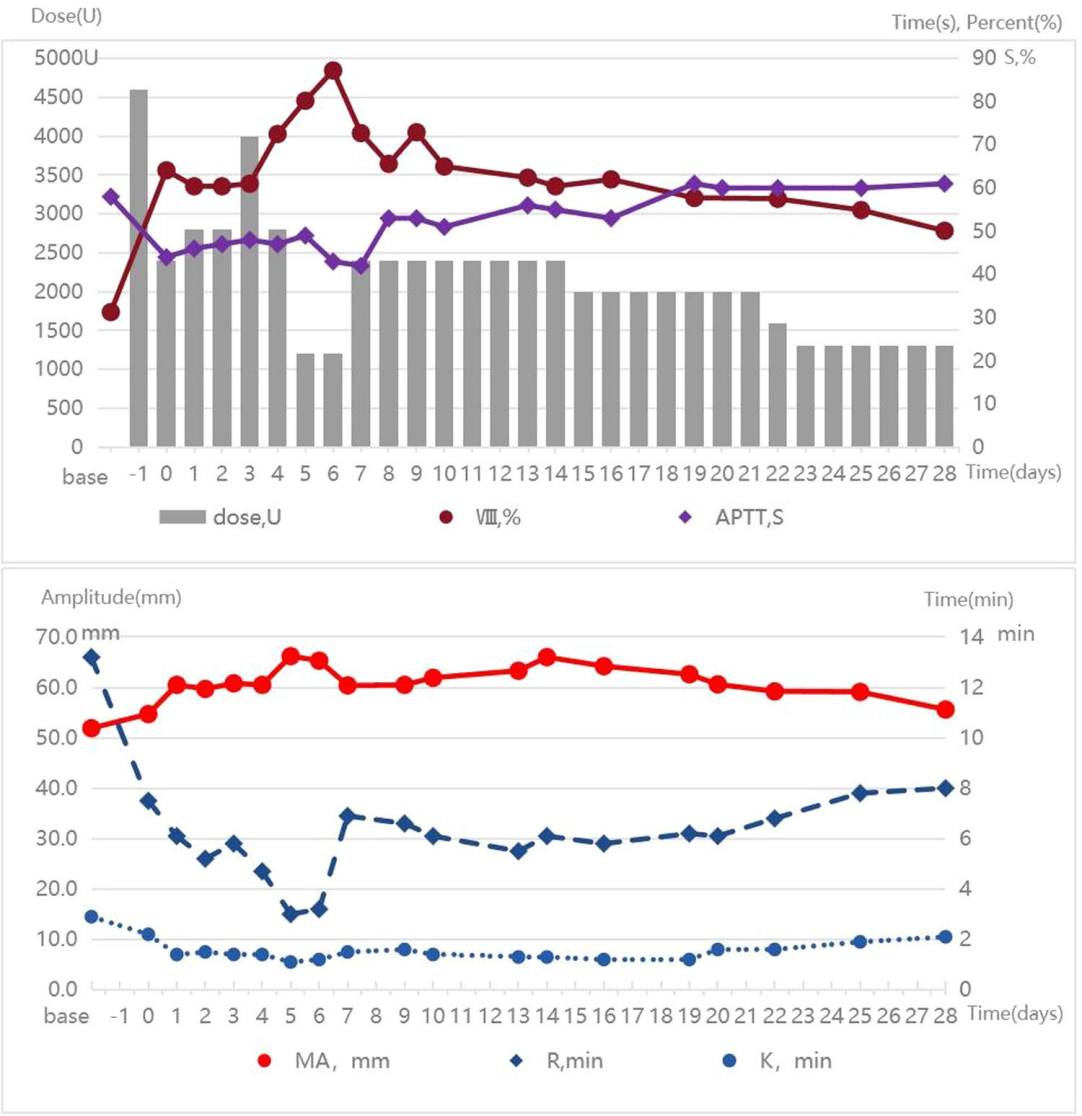
Trends of dose of coagulation factor VIII and laboratory results of factor VIII activity, APTT and TEG from 1 day pre to 28 days after surgery.

**Figure 4:**
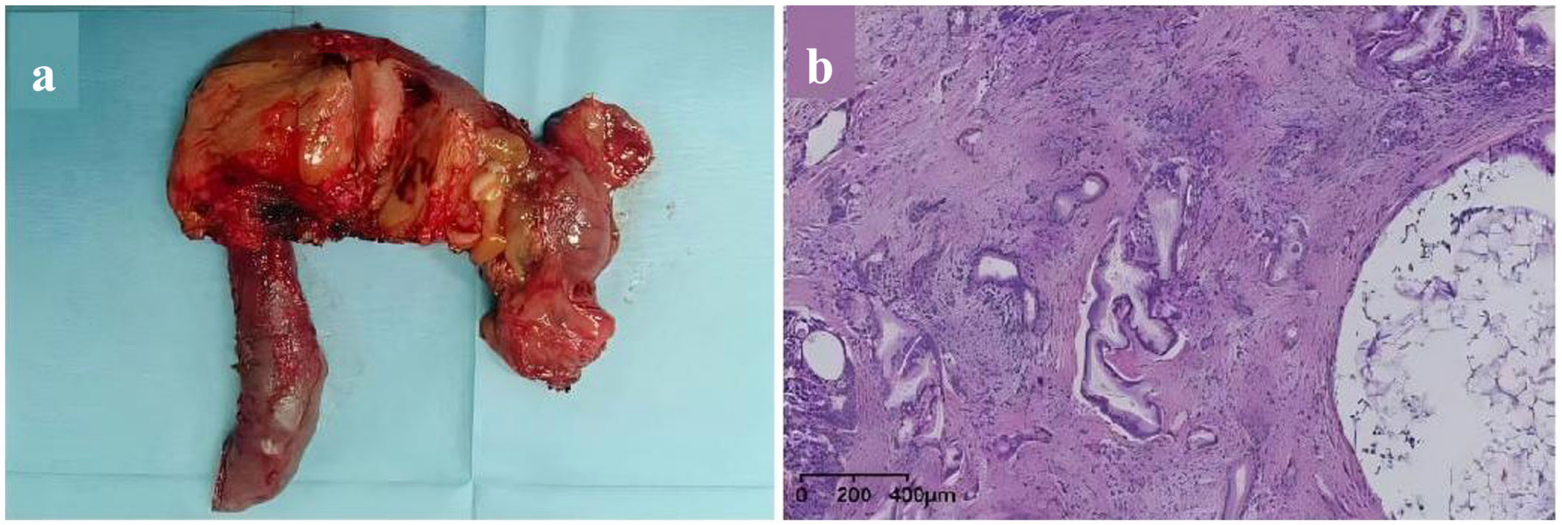
(a) Surgical resection of tumor gross specimen. (b) Pathological examination was a moderately differentiated adenocarcinoma.

The patient was monitored in the ICU postoperatively for close surveillance and enhanced treatment. From Day 1 to Day 28 after surgery, the infusion dose of coagulation factor VIII or cryoprecipitate was regularly adjusted based on the quantity of abdominal drainage fluid, laboratory results of hemoglobin, activation of partial thromboplastin time, thromboelastography, coagulation factor activity, and coagulation factor inhibitors (always negative). The specific results are shown in Figure 3. The tumor is situated in the pancreatic head, with dimensions of 3 cm × 3 cm × 2.5 cm, and there is evidence of necrosis within the tumor (Figure 4a). Pathological examination was revealed a moderately differentiated adenocarcinoma (Figure 4b). Normal bowel function resumed by postoperative Day 2, and oral intake began on postoperative Day 3. The drainage tube at the bile intestinal anastomosis and the pancreatic intestinal anastomosis were removed on postoperative Days 7 and 14, respectively. He recovered well without severe complications, and chemotherapy for pancreatic cancer was successfully started on Day 42.

## 3 Discussion

In this intricate case of an 82‐year‐old patient diagnosed with pancreatic head cancer and HA, the risk of postoperative mortality presented a formidable challenge. The SMC model, characterized by a smaller, highly focused team including a surgeon and an internist offered a tailored and efficient solution to navigate this complex scenario. This dedicated collaboration is well‐suited for the management of complex surgical cases such as the case presented here.

The patient's advanced age added to the complexity of the case. As the incidence of malignancy increases with age, pancreatic head carcinoma is increasingly identified in the older brackets of the population [6]. Whether to execute PD or not for older people could pose a dilemma, especially for those aged over 80 years and SMC teams should be utilized in these complex situations. The SMC team implemented many perioperative optimization approaches for this patient to avoid complications and to increase patient satisfaction. The surgeons and internists together performed a detailed history and preoperative examination, allowing them to diagnose and optimize patient comorbidities pre‐ and postoperatively, and avoid acute medical decompensation to facilitate a safe recovery.

Another of the most challenging aspects of this case was the risk of significant intraoperative or postoperative bleeding. Due to the dissection around important vessels and three complex reconstructions following extensive dissection, PD can be associated with massive blood loss in select cases [1, 2, 6]. Moreover, the history of cholecystectomy in this case increased the difficulty of the surgery and the risk of intraoperative bleeding. We choose the RPD because robotic surgical system advancements contribute to the flexibility and stability of surgeons during surgery [7], which is beneficial for shortening surgical time, reducing trauma, and blood loss. Pancreatic fistula is widely regarded as the most common cause of significant bleeding after PD [8]. We took the following measures to reduce pancreatic fistula: (1) the internal stent was placed into the main pancreatic duct and the intestine to avoid ischemic necrosis of the main pancreatic duct; (2) two figure‐eight suture lines spanning the entire pancreas were placed to close the opening of pancreatic ducts in small branches of the pancreas; (3) a single layer continuous pancreatic suture was used to lift the jejunal wall to completely cover the pancreatic section, which can reduce pancreatic leakage through the needle hole. As expected, this patient did not experience pancreatic fistula after surgery.

It must be noted that patients with HA undergoing surgery often can experience life‐threatening bleeding with the development of inhibitory antibodies against the infused factor VIII [9]. Previous research has found that major risk factors for inhibitors are surgical procedures, older age, and intensive exposure to factor VIII [10]. The only of these risk factors that can be addressed is to reduce the dose of factor VIII. Therefore, we minimized the dose of factor VIII to maintain its activity within the range of 60%–80%, which can avoid bleeding events caused by insufficient coagulation factors and reduce the risk of inhibitors. Our expected results were achieved, and the patient did not experience any bleeding and inhibition of factor VIII.

## 4 Conclusion

The SMC team can implement many optimization methods for complex patients to avoid severe complications. This strategy supports a shift from passive conservative treatment to active and preventive treatment and warrants broader implementation in high‐risk surgical settings.

## Author Contributions

**Wenning Lu:** conceptualization (equal), data curation (equal), formal analysis (equal), investigation (equal), methodology (equal), writing – original draft (lead), writing – review and editing (lead). **Chaoyang Liu:** data curation (equal), investigation (equal). **Jing He:** data curation (equal), investigation (equal). **Rui Cheng:** conceptualization (lead), writing – review and editing (lead).

## Ethics Statement

The study protocol was approved by the Ethics Committee of the PLAGH(S2022‐664‐01), and the procedures conform to the principles of the Declaration of Helsinki.

## Consent

The patient provided written informed consent at the time of entering this study.

## Conflicts of Interest

The authors declare no conflicts of interest.

## Data Availability

The data that support the findings of this study are available on request from the corresponding author. The data are not publicly available due to privacy or ethical restrictions.
